# Clinical heterogeneity of human parvovirus B19 infection following adult liver transplantation

**DOI:** 10.1097/MD.0000000000012074

**Published:** 2018-08-24

**Authors:** Jiabin Zhang, Bo Ren, Ren Hui, Yanling Sun, Zhenwen Liu, Shaotang Zhou

**Affiliations:** a Center of Hepatopancreaticobiliary Surgery and Liver Transplantation; b Anesthetic Department, 302 Hospital, Beijing, China.

**Keywords:** anemia, heterogeneity, human parvovirus B19, liver transplantation

## Abstract

Severe aplastic anemia and its secondary comorbidities associated with human parvovirus B19 infection is a rare and sometimes refractory complication following liver transplantation.

We retrospectively reviewed data for 217 adult liver transplant recipients from donations after death in China March 2013 through May 2017, 5 patients with human parvovirus B19 infectious diseases were teased out, and diagnoses were made from positive serological marker, bone marrow aspiration, and genome assay, other hemolytic causes were excluded. Severe aplastic anemia and its comorbidities were confirmed, combination of immunoglobulin and blood transfusion as well as immunosuppressant switch was employed for 5 recipients.

Four male and 1 female recipients were diagnosed with human parvovirus B19 infections based on clinical presentations, bone marrow aspiration, and nested PCR, age ranged from 47 to 62 years, the onset time from liver transplantation varied from 29 to 415 days, anemia improved in 5 patients, 2 deaths occurred due to parvovirus-related morbidities, 1 patient died from de novo carcinoma of the tongue 2 years later and unrelated to parvovirus, 2 other recipients are still alive.

Human parvovirus B19 infectious disease is a rare but clinically significant infection whose comorbidities will bring about more attentions.

## 1 Introduction

Parvovirus B19 virus (B19 V) was discovered unexpectedly in 1974 and later known to be pathogenic in humans. This virus is ubiquitous and can infect immune-competent and immune-compromised people; its manifestations depend on the immunologic and hematologic status of the host.^[1]^ In 1986, Starzl first published B19 V infection after pediatric liver transplantation,^[2]^ afterwards, B19 V infections after solid-organ transplantation and hematopoietic stem cell transplantation have been increasingly reported.^[3,4]^ Aplastic anemia is the significant clinical presentation and easily improved under the immunoglobulin infusion and supportive treatments. However, secondary conditions including hepatitis, pneumonitis, myocarditis, and allograft dysfunction will likely develop and rarely reported, especially in immune-compromised recipients.^[1,5]^ Herein we reported 5 cases of liver transplant recipients with human -B19 V infectious diseases from donations after death in China, clinical outcomes were heterogeneous.

## 2 Patients and methods

We retrospectively reviewed 217 adult liver transplantations from donations after death performed March 2010 through May 2017 in the liver center of hospital 302. The indications for LT were end-stage liver diseases and/or hepatocellular carcinoma. Patients with malignancies who underwent liver transplantation met the Milan criteria. For the peri-operational management and follow-up, any recipient with very low hemoglobin would undertake the laboratory tests including reticulocyte count, iron studies, serum folate and Vitamin B12 levels, liver and renal functions, and occult stool hematest. All patients had been assayed to determine the presence of cytomegalovirus, Epstein–Barr virus, herpes virus, and B19 V. Ultrasound and x-ray were performed routinely to monitor the patients. Hemolytic tests and bone marrow aspirate were done to evaluate anemic state. For basic immunosuppression, it consisted of tacrolimus and mycophenolate mofetil. Combination of nucleotide analogue and HBV immunoglobulin was used indefinitely for prophylaxis of HBV recurrence. When B19 V infection was suspected, serum antibody was tested first, quantified PCR was conducted to confirm the diagnosis, once confirmed, 300 mg/kg of immunoglobulin was infused daily for 7 to 10 days, erythropoietin complement and blood transfusion was administered, tacrolimus was switched to cyclosporine A.

We obtained ethical approval from the Committee of Ethics in 302 Hospital. Informed consent was obtained from recipients or their family. All procedures followed were in accordance with the ethical standards of responsible committee on human experimentation (institutional and national) and with the Helsinki Declaration of 1964 and later versions.

## 3 Results

**Figure 1:**
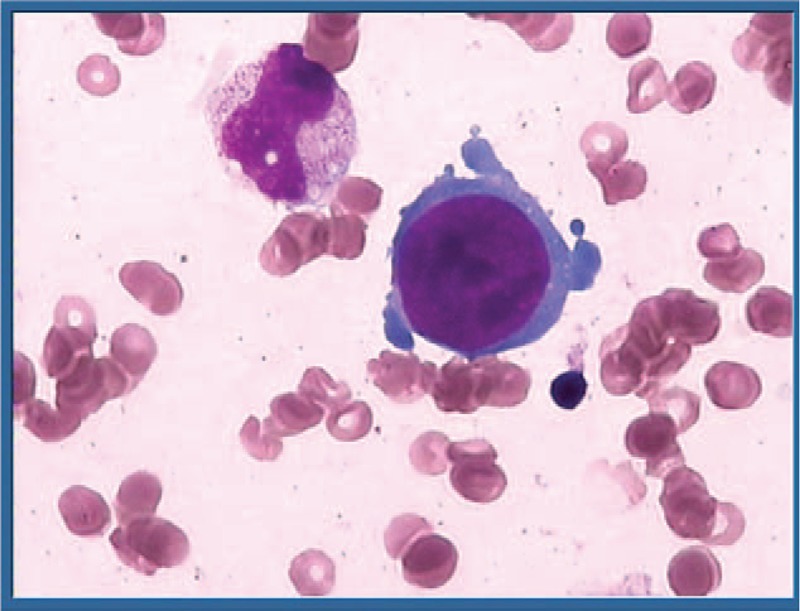
Decreased erythroid precursors shown in bone marrow aspirate smear (Wright–Giemsa stain).

Four male and 1 female recipients with severe anemia were infected with B19 V, which were clinically confirmed by seropositive Ig M, DNA quantification and bone marrow aspiration. They were aged 47 to 62 years (Table 1). Severe anemia occurred in 5 patients with various leukopenia and thrombocytopenia (Fig. 1); B19 V-related comorbidities implicated in 3 patients. Patient 1 presented with high fever, severe anemia in 29 days after LT, DNA level of B19 iV was 4.11 × 10^3^ IU/mL, combined treatment of immunoglobulin and immunosuppressant switch started immediately on diagnosis, HBV viruses replicated with higher HBV DNA level and original hepatocellular carcinoma recurred, those factors brought about liver dysfunctions and his situations progressed rapidly, as a result this patient died of cachexia derived from anemia and cancer progression 19 days after diagnosis. Patient 2 developed B19 V infection over 1 year after LT, he suffered from moderate fever a few days, laboratory test revealed that he was in a state of immunodeficiency of lymphocyte B, his B19 V DNA level was 1.07 × 10^2^ IU/mL, after immunoglobulin infusion and symptomatic treatment for 2 weeks, he recovered from this episode; 2 years later de novo carcinoma of the tongue was detected and caused his death. Patient 3, 5 had moderate fever and only pure red cell aplasia, almost no other morbidities existed, and quantitative PCR revealed that B19 V DNA load was 2.14 × 10^3^, 1.95 × 10^2^ IU/mL respectively, both recovered after immunoglobulin infusion and symptomatic treatment and have been doing well. Patient 4 developed this infection on day 67 post-operation and progressed rapidly, her B19 V DNA load was 7.41 × 10^6^ IU/mL, though combined treatment started timely, hepatitis and pneumonia took place, auto-immune hepatitis recurred, co-infection of mycoplasma and chlamydia complicated of B19 V infection, timely combined treatment, and supportive care was applied, and she died of its comorbidities 30 days after the diagnosis of B19 V infection.

**Table 1:**
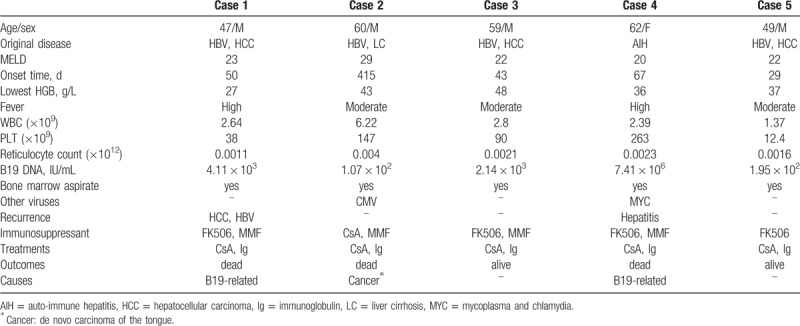
Characteristic of B19 viral infections in 5 recipients.

## 4 Discussion

B19 V is a small non-enveloped single-stranded DNA virus which can infect most people at all ages, people infected withB19 V can produce specific antibodies, IgM first and IgG later, IgG can persist lifelong and protect against re-infection, the prevalence of IgG is on the rise over time in the general population, ranging 30% to 80% in adults, whereas its viremia or presence of viral DNA is rare, clinically its infection is asymptomatic or has only mild, non-specific symptoms.^[1,4]^ In immunocompromised hosts unable to neutralize antibody, B19 V can replicate in erythrocyte precursors efficiently and preferentially, this infection may persist and lead to pure red cell anemia or even comorbidities.^[3,6]^ Atypical giant pronormoblasts in bone marrow or peripheral blood is suggestive of the presence of B19 V, which can be detected in serum by indirect assays and direct isolation of viral genome.^[7]^ The diagnosis of B19 V infection was based on its seropositive marker and bone marrow aspiration, DNA positivity while other hemolytic causes are excluded.

The combined treatment of immunoglobulin infusion and symptomatic treatments including blood transfusion is effectively curative to neutralize B19 viruses and improve anemia, especially only pure red cell aplastic anemia as reported in the literature.^[8]^ We addressed heterogeneity of B19 V infections in liver transplant recipients. This infection occurred earlier after transplantation, at that time, recipients needed to be patently immunosuppressed for avoidance of rejection, interplay of underlying immunosuppressed state, anemia and its concomitant leukocytes and other viruses could surely bring about complicated conditions, organ-invasive disorders including hepatitis, pneumonia, carditis, or co-infections with other viruses, bacteria or pathogens, will probably ensue, this comorbidities influence treatment options and sometimes lead to poor outcomes. It was reported that common co-infection of HBV and B19 V likely progressed to more severe hepatitis B-associated liver disease in the General HBV population,^[9]^ this differs from immune status whereas our reported cases are immunocompromised and combination of nucleotide analogue and HBV immunoglobulin was used indefinitely for prophylaxis of HBV recurrence. Albert et al^[3]^ searched 9 liver transplant recipients infected with B19V with 1 recipient death in literature. Ming et al^[10]^ reported that the outcomes of 12 adult recipients were favorable with B19 V infections, only pure red cell aplastic anemia due to B19 V infection presented clinically and improved easily, their underlying diseases were not narrative. Lee et al^[11]^ reported a case that a recipient with hepatocellular carcinoma was fully recovered from B19V-related pure red cell aplastic anemia 2 months after liver transplantation, Annelie et al^[12]^ documented that low-level DNAemia of parvovirus B19 in adult transplant recipients is not associated with anaemia. In our report, 5 recipients presented severe anemia, pure red cell aplasia was amenable to cure in 3 patients, while prognosis was poor in 2 patients due to this viral infectious disease complicated by organ-invasive disorders or co-infections. From above mentioned, a wide spectrum of B19 V infection can present clinically and heterogeneously, only pure red cell aplasia could improve easily, organ-invasive disorder involved in B19 V infection is refractory and death will likely occur. As LT is more common, there is a need to better understand the interplay between B19 V infection and other viruses as well as its comorbidities.

Transmission of B19 V infection occurs with 3 types: via the respiratory route, through blood-derived products, and administered parenterally and vertically.^[1]^ The sources of B19 V in recipients come from three types. First, viruses may be from donor. In China, donation after death is a unique clinical practice that is donation after brain death followed by circulatory death, the potential donors stayed in intensive care unit longer; they are at higher risk of B19 V infection. Second, because non-enveloped viruses such as B19 V cannot be efficiently removed by conventional sterile methods in the processing of blood and its products including solvent/detergent treatment, pasteurization, dry heating and filtration which therefore pose a potential risk to contaminate the blood or blood products, they are extensively utilized in transplantation. It was reported that B19 V DNA or viruses were detected in 54.2% of plasma pools and recently produced blood products including immunoglobulin, factor VIII, fibrinogen and prothrombin complex concentrate, not including albumin in China.^[13]^ Third, the recipients were infected with B19 V perioperatively, which reactivated in the early post-transplant days and can be pathogenic. Hitherto nosocomial B19 V infection maybe the main reason for those recipients.^[14,15]^ AASLD guideline for transplantation set that organ donor screen for B19 V should be considered prior to transplantation.^[16]^ No guideline of organ or blood donor screening for this virus is available in most countries including China to date. And as of today, no approved human vaccine protects against B19 V.

The main limitation of this study was retrospective study with fewer cases. Our concern is to address the significances clinical heterogeneities caused by B19 V infection.

## 5 Conclusion

B19 V infection is a disease with a heterogeneous prognosis following adult liver transplantation and sometimes brings about more concerns.

## Author contributions

JZ wrote the draft, BR revised the draft, HR collected the data, YS collected references, ZL performed surgeries, SZ conceived, designed, and finalized the study.

**Conceptualization:** Shaotang Zhou.

**Data curation:** Ren Hui.

**Validation:** Shaotang Zhou, Zhenwen Liu, Bo Ren.

**Visualization:** Yanling Sun.

**Writing – original draft:** Jiabin Zhang.
